# Interactions Between *Pseudomonas* Immunotoxins and the Plasma Membrane: Implications for CAT-8015 Immunotoxin Therapy

**DOI:** 10.3389/fonc.2018.00553

**Published:** 2018-11-27

**Authors:** Monika Bokori-Brown, Jeremy Metz, Peter G. Petrov, Francis Mussai, Carmela De Santo, Neil J. Smart, Sarah Saunders, Bridget Knight, Ira Pastan, Richard W. Titball, C. Peter Winlove

**Affiliations:** ^1^College of Life and Environmental Sciences, School of Biosciences, University of Exeter, Exeter, United Kingdom; ^2^College of Engineering, Mathematics and Physical Sciences, Department of Physics and Astronomy, University of Exeter, Exeter, United Kingdom; ^3^Institute of Immunology and Immunotherapy, College of Medical and Dental Sciences, University of Birmingham, Birmingham, United Kingdom; ^4^Exeter Surgical Health Services Research Unit, Royal Devon and Exeter Hospital, Exeter, United Kingdom; ^5^Histopathology Department, Royal Devon and Exeter Hospital, Exeter, United Kingdom; ^6^National Institute for Health Research Exeter Clinical Research Facility, Royal Devon and Exeter National Health Service Foundation Trust, Exeter, United Kingdom; ^7^Laboratory of Molecular Biology, Center for Cancer Research, National Cancer Institute, NIH, Bethesda, MD, United States

**Keywords:** immunotoxin, cancer treatment, pediatric acute lymphoblastic leukemia, CAT-8015, Moxetumomab pasudotox, red blood cells, VLS, HUS

## Abstract

Acute Lymphoblastic Leukemia (ALL) remains the most frequent cause of cancer-related mortality in children and novel therapies are needed for the treatment of relapsed/refractory childhood ALL. One approach is the targeting of ALL blasts with the *Pseudomonas* immunotoxin CAT-8015. Although CAT-8015 has potent anti-leukemia activity, with a 32% objective response rate in a phase 1 study of childhood ALL, haemolytic-uremic syndrome (HUS) and vascular leak syndrome (VLS), major dose-limiting toxicities, have limited the use of this therapeutic approach in children. Investigations into the pathogenesis of CAT-8015-induced HUS/VLS are hindered by the lack of an adequate model system that replicates clinical manifestations, but damage to vascular endothelial cells (ECs) and blood cells are believed to be major initiating factors in both syndromes. Since there is little evidence that murine models replicate human HUS/VLS, and CAT-8015-induced HUS/VLS predominantly affects children, we developed human models and used novel methodologies to investigate CAT-8015 interactions with red blood cells (RBCs) from pediatric ALL patients and ECs of excised human mesenteric arteries. We provide evidence that CAT-8015 directly interacts with RBCs, mediated by *Pseudomonas* toxin. We also show correlation between the electrical properties of the RBC membrane and RBC susceptibility to CAT-8015-induced lysis, which may have clinical implication. Finally, we provide evidence that CAT-8015 is directly cytototoxic to ECs of excised human mesenteric arteries. In conclusion, the human models we developed constitutes the first, and very important, step in understanding the origins of HUS/VLS in immunotoxin therapy and will allow further investigations of HUS/VLS pathogenesis.

## Key points

- Development of human models to investigate HUS/VLS pathogenesis.
- *Pseudomonas* immunotoxins directly interact with red blood cells, mediated by the toxin component PE38, a novel mechanism for atypical HUS
- New biophysical tools are provided to study atypical HUS caused by other toxins and immunotoxins.

## Introduction

Acute Lymphoblastic Leukemia (ALL) is the most common malignancy of childhood (1) and it remains the most frequent cause of cancer-related mortality in children (2). The survival benefit of conventional chemotherapy in the treatment of multiply relapsed and chemotherapy-refractory pediatric ALL has plateaued and novel approaches have recently undergone early phase clinical trials to overcome these limitations (3). One approach is the targeting of ALL blasts with CAT-8015 (HA22, Moxetumomab pasudotox), a second-generation, high affinity recombinant immunotoxin (IT) composed of a 38 kDa fragment of *Pseudomonas* exotoxin A (PE38) fused to the disulphide-linked variable fragment of the murine anti-CD22 monoclonal antibody RFB4, which replaces the toxin's native cell binding domain I to specifically target CD22 antigen on the surface of ALL lymphoblasts (4–7). On antigen binding, CAT-8015 is rapidly internalized (8), traffics through the cell and undergoes several processing steps before the toxin enters into the cytosol, where it inhibits protein synthesis, leading to apoptotic death of the cancer cells (9).

CAT-8015 has potent anti-leukemia activity, with an objective response rate of 32% in a phase 1 clinical trial of relapsed/refractory pediatric ALL (3). However, haemolytic-uremic syndrome (HUS) and vascular leak syndrome (VLS), toxic and dose-limiting side effects, have limited the use of this therapeutic approach in children [HUS and VLS occurring in 13 and 7% of patients, respectively (3)], despite its recent FDA approval for the treatment of relapsed/refractory adult hairy cell leukemia, with an objective response rate of 75% (HUS and VLS occurring in 5 and 2.5% of patients, respectively) (10). These side effects do not affect all patients [10–15% are affected (3)] and there is evidence that interpatient variability is physiological rather than genetic in origin (11). Due to its persistent toxicity profile, prophylactic treatment with the corticosteroid dexamethasone was required in subsequent Phase 2 studies of childhood ALL.

HUS, characterized by intravascular haemolysis, thrombocytopenia, microvascular thrombosis and acute kidney failure (11, 12), is grouped into three forms: (1) typical HUS, the most frequent form of HUS caused by Shiga toxin-producing *Escherichia coli* infection, (2) atypical HUS, largely associated with an overactive complement system, and (3) secondary HUS, associated with a coexisting disease or trigger, such as pediatric ALL (13) or CAT-8015 cancer therapy (11, 14). VLS is characterized by increased vascular permeability, accompanied by extravasation of fluids and proteins, leading to interstitial oedema, and in severe cases, pulmonary and cardiovascular failure (15).

The mechanisms responsible for CAT-8015-induced HUS/VLS are poorly understood but nonspecific damage to vascular endothelial cells (ECs) (15, 16) and blood cells (3, 11) are believed to be major initiating factors in both syndromes, with no evidence for the involvement of the complement system. The clinically significant effects of the interactions of CAT-8015 with blood cells are likely to be through red blood cell (RBC) haemolysis, as evidenced by decreased hemoglobin being one of the most common treatment-emergent adverse events in a phase 1 study of childhood ALL (3).

Investigations into the pathogenesis of HUS/VLS are complicated by the lack of an adequate model system that closely corresponds to clinical manifestations. Our current understanding of the pathogenesis of HUS/VLS is largely based on the use of cultured ECs (17, 18) or animal models (19). However, cultured ECs are unphysiological, and studies of vascular permeability to water and solutes, changes in which are likely to be key with ITs, are unrepresentative of those in intact vessels (20, 21). Animal models may not be relevant to HUS/VLS in humans either, because of differences between animal and human vasculature (15, 19). With the exception of a rat model (19), animal models for *Pseudomonas* IT-induced HUS/VLS are also lacking, since studies of non-human primate (22, 23) and murine (24) models have failed to provide new insights into human VLS/HUS. Since there is little evidence that murine models replicate human HUS/VLS, and CAT-8015-induced HUS/VLS is predominantly seen in children, we developed human models and used novel methodologies to investigate CAT-8015 interactions with red blood cells (RBCs) from pediatric ALL patients and ECs of excised human mesenteric arteries.

The physical properties of the plasma membrane, such as its electrostatic status, are indicators of lipid composition and dynamics, changes in which reveals interaction of molecules with the membrane (25, 26). We previously reported that the physical properties of the plasma membrane can also influence cell susceptibility to protein toxins (27). Therefore, we investigated the interactions of CAT-8015 and its components with RBCs using a number of sensitive biophysical measurements, such as membrane dipole potential (MDP) measurements, which detect subtle changes in the electrical properties of the membrane, and analysis of the absorption of 415 nm light by hemoglobin, which detects subtle changes in cell morphology. All measurements were made at single cell level with the ultimate aim to understand interpatient variability in response to CAT-8015 treatment.

We provide evidence that CAT-8015 directly interacts with RBCs, mediated by its toxin component PE38. We also show correlation between the electrical properties of the RBC membrane and the susceptibility of RBCs to CAT-8015-induced haemolysis, which could identify ALL patients who will most benefit from CAT-8015 immunotoxin therapy. Finally, we provide evidence that CAT-8015 is directly cytototoxic to ECs of excised human mesenteric arteries.

## Materials and methods

### Materials

Chemicals were purchased from Sigma, UK, unless otherwise stated. CAT-8015 was provided by MedImmune (Gaithersburg, MD, USA) (28). The concentration of CAT-8015 used throughout this study (500 ng/ml) corresponds to the plasma level achieved in pediatric patients with ALL treated at the upper dose levels in Phase I trials of CAT-8015 (range 311-586 ng/ml) (3). RFB4 mouse monoclonal antibody (full anti-CD22 antibody) and SS1P [a *Pseudomonas* IT targeting mesothelin-expressing solid tumours (29)] were provided by Ira Pastan.

### Ethics statement

This study was carried out in accordance with the recommendations of the South Central-Hampshire Regional Ethics Committee and complies fully with the Standard Operating Procedures for Research Ethics Committees in the UK. The protocol was approved by the South Central-Hampshire Regional Ethics Committee (Ref: 10/H0501/39) and NRES Committee South West–Central Bristol (Ref: 16/SW/0056). All subjects gave written informed consent in accordance with the Declaration of Helsinki.

### Patient samples

Fresh whole blood from paediatric ALL patients and age-matched healthy individuals were collected by venipuncture at Birmingham Children's Hospital into heparin coated tubes. ALL patient samples were consented for immune markers by ethics, Ref: 10/H0501/39, by a UK Regional Ethics Committee. Mesentery tissues were obtained from patients undergoing routine colorectal surgery. Recruitment and sample collection was facilitated via the ethically approved Royal Devon and Exeter Tissue Bank (RDETB, REC no: 16/SW/0056) and the National Institute for Health Research Exeter Clinical Research Facility. Application to collect and process anonymized mesentery samples was approved by the RDETB Steering committee. Following surgical resection, the samples were immediately transferred to the Royal Devon & Exeter Histopathology Department for removal of a sample of mesentery tissue furthest from any tumor. The sample was then transported on ice in DPBS (Dulbecco's Phosphate Buffered Saline) buffer without calcium and magnesium, pH 7.0–7.2, supplemented with 1 mg/ml bovine serum albumin (BSA; DPBS/BSA) in a sealed polystyrene box for immediate processing and analysis.

### Preparation of red blood cells

Fresh whole blood in heparin coated tubes was centrifuged at 1,000 × *g* for 10 min at room temperature to separate red blood cells (RBCs) from plasma. After careful removal of plasma, packed RBCs (100 μl) were resuspended in 1 ml DPBS without calcium and magnesium, pH 7.0–7.2 (Invitrogen) supplemented with 1 mg/ml bovine serum albumin (BSA; DPBS/BSA) and washed three times with 1 ml DPBS/BSA. Each washing step with 10-fold volume of DPBS/BSA is expected to achieve >10-fold dilution of plasma proteins in already plasma separated RBCs to remove complement. Finally, 1.5 μl of extensively washed RBCs was resuspended in 1 ml DPBS/BSA for incubations with immunotoxin. Removal of complement was confirmed by the immunoturbidimetric assays for C4 and C3c on Roche cobas®8000 system. Any residual complement that may remain cannot be activated due to the lack of divalent ions in the resuspension buffer.

### Measurement of the MDP

Measurement of the membrane dipole potential (MDP) was performed in μ-Slide I^0.2^ Luer poly-L-lysine imaging chambers (Ibidi), as described previously (27). In brief, 5 μl of 1 mg/ml Di-8-ANEPPS (Thermo Fisher) in ethanol was added to 1 ml RBC suspension at a concentration of 3 × 10^7^ cells/ ml in DPBS/BSA, and c000000000ells were incubated at 37°C for 1 h. Subsequently, cells were washed three times in DPBS/BSA to remove excess dye and resuspended in 1 ml DPBS/BSA with or without CAT-8015 (500 ng/ml) for further incubation at 37°C for 1 h. Ratiometric fluorescence images were captured by an Olympus IX50 inverted microscope (Olympus Optical, Hamburg, Germany) equipped with a Plan-Neofluar 63 × /1.25 oil immersion objective, an AVT Stingray F-145B camera and Live Acquisition 2.2.0.8 software.

### Measurement of RBC morphology

To monitor CAT-8015-induced changes in RBC morphology, Di-8-ANEPPS-labeled RBCs from ALL patients were imaged before and after exposure to buffer only (DPBS/BSA) or buffer containing CAT-8015 (500 ng/ml) for 1 h at room temperature in a μ-Slide I^0.2^ Luer poly-L-lysine imaging chamber (Ibidi). Images were captured by an Olympus IX50 inverted microscope (Olympus Optical, Hamburg, Germany) equipped with a 415 nm diode light source (ThorLabs), an Olympus UPlanSApo 60 × /1.20W objective, an AVT Stingray F-145B camera and Live Acquisition 2.2.0.8 software.

The radial profile of individual cells was determined by a custom Python script (30–32). In brief, approximate cell sizes were detected by the strongest peaks in scale-space (33) and used as an input to the background subtraction algorithm, which is a port of ImageJ's rolling-ball algorithm (34). Cells were then detected as regions of intensity higher than a certain threshold value, determined using an Otsu's method, resulting in a mask image. A marker-based watershed transform on the distance transform of the mask image was used to delineate touching cells. Next, we applied several morphological constraints to the cell regions, such as size filtering, to remove any that were too small or too large. The final cell regions were used to generate the radial intensity profiles by averaging the intensity (minus the cell's local background value) at each radial position.

### Correlation between the MDP of RBCs and their susceptibility to CAT-8015-induced lysis

To investigate whether differences in the MDP of individual cells within a population of RBCs are correlated with their susceptibility to CAT-8015-induced lysis, we performed ratiometric fluorescence imaging of a number of individual cells within a population of RBCs from ALL patients before CAT-8015 treatment, as described in “Measurement of the MDP.” The buffer (DPBS/BSA) surrounding the cells was then exchanged with buffer containing CAT-8015 (500 ng/ml) and after 1 h incubation at room temperature RBCs were exposed to 415 nm light to monitor cell lysis, as described in “Measurement of RBC morphology.”.

### Measurement of vascular permeability

To investigate the effect of CAT-8015 on vascular permeability, excised human mesenteric arteries were cut into 0.5 cm transverse sections, washed three times with 10 ml DPBS without calcium and magnesium, pH 7.0–7.2 (Invitrogen), and incubated with buffer only (DPBS) or buffer containing CAT-8015 (500 ng/ml) in the presence of the vascular permeability marker BSA labeled with the red fluorophore Alexa Fluor™ 594 (Thermo Fisher; Alexa594-BSA). After incubation for 1 h at 37°C, arteries were washed three times with DPBS, frozen in OCT embedding medium (Tissue-Tek, Electron Microscopy Sciences), sectioned as 10 μm transverse sections using a Leica microtome (Leica Microsystems, Milton Keynes, UK) and imaged using a Leica SPF5 confocal microscope (Leica, Heidelberg, Germany) equipped with a HCX PL APO lambda blue 63 × /1.4 oil immersion lens, LAS AF Version 1.4.0 Build 613 software and Leica DFC Camera. Excitation light was provided by the 594 nm line of a helium neon laser.

To quantify the effect of CAT-8015 on vascular permeability, images were manually segmented using ImageJ software (34) to label the lumen and intima regions. These labeled masks and corresponding original Alexa594 fluorescence data were then processed by a Python script (30–32), which loaded the images and performed a distance transform on the labeled region to determine the distance of each pixel in the intima from the lumen. The mean fluorescence value at each distance (i.e., the mean intensity profile) was then calculated by using these distances and the loaded fluorescence values.

### Transmission electron microscopy

For transmission electron microscopy (TEM) studies, excised human mesenteric arteries were cut into 0.5 cm transverse sections, washed three times with 10 ml DPBS without calcium and magnesium, pH 7.0–7.2 (Invitrogen), and incubated with buffer only (DPBS) or buffer containing CAT-8015 (500 ng/ml). After incubation for 1 h at 37°C, tissues were fixed in 2% glutaraldehyde and 2% paraformaldehyde in 0.1 M PIPES (1,4-Piperazinediethanesulfonic acid) buffer (pH 7.2). After removal of excess fat tissue the artery sections were sliced into 1 mm pieces, washed with buffer three times and post-fixed for 1 h in 1% osmium tetroxide (reduced with 1.5 % wt/vol potassium ferrocyanide) in 0.1 M sodium cacodylate buffer (pH 7.2). The samples were then washed in deionized water three times and incubated with 1% aqueous uranyl acetate for 30 min (en-bloc staining). After washes in deionized water three times the samples were dehydrated through a graded ethanol series and subsequently embedded in Spurr resin (TAAB Laboratories, Aldermaston, UK). 70 nm ultrathin sections were produced using a Leica EM UC7 ultramicrotome (Leica Microsystems, Milton Keynes, UK) and collected on pioloform-coated copper slot EM grids (Agar Scientific, Stansted, UK). Some of the grids were contrasted with lead citrate before imaging. The sections were analyzed using a JEOL JEM 1,400 transmission electron microscope operated at 120 kV and images taken with a digital camera (ES 100W CCD, Gatan, Abingdon, UK).

### Histological tissue preparation

Excised human mesenteric arteries were cut into 0.5 cm transverse sections, washed three times with 10 ml DPBS without calcium and magnesium, pH 7.0–7.2 (Invitrogen), and incubated with buffer only (DPBS) or buffer containing CAT-8015 (500 ng/ml). After incubation for 1 h at 37°C, tissues were fixed in 10% buffered paraffin and processed to paraffin blocks using standard laboratory methods. Tissues were sectioned as 6 μm transverse sections using a Leica microtome (Leica Microsystems, Milton Keynes, UK) and routine hematoxylin-and-eosin (H&E) stains were applied as per standard laboratory protocols. Ets-Related-Gene (ERG) immunohistochemistry as an endothelial marker was performed on paraffin sections as per standard laboratory protocols. Histological sections were imaged using an Olympus BX53 microscope (Olympus Optical, Hamburg, Germany) equipped with a UPlanFL N 40 × /0.75 objective and OLYMPUS cellSens Entry 1.16 software.

### Statistics

Data were analyzed using Prism v7 software (GraphPad Software, Inc., La Jolla, CA, USA). A *P*-value < 0.05 was considered significant. Data are expressed as the mean value ± SEM.

## Results

### CAT-8015-induced changes in the MDP of the RBC plasma membrane

**Figure 1:**
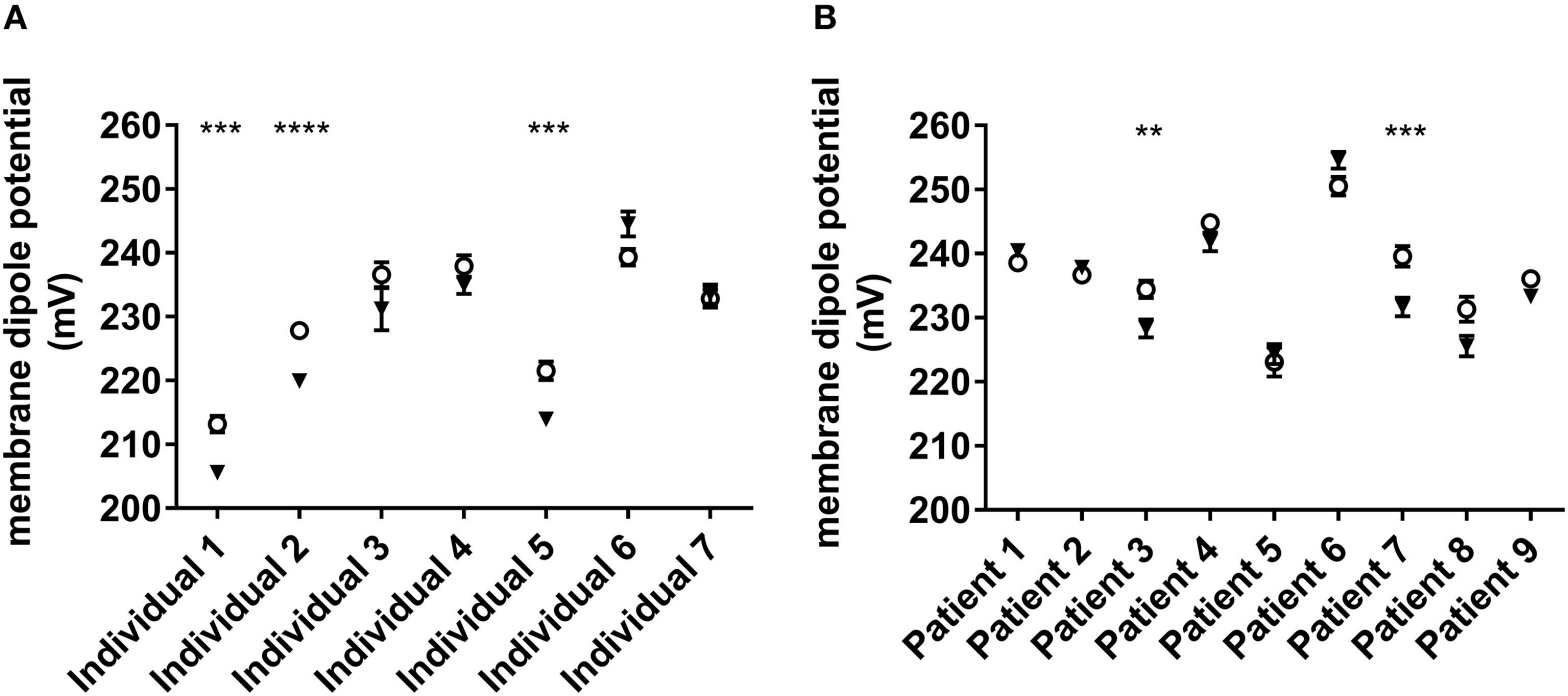
CAT-8015-induced changes in the MDP of RBCs. **(A)** Effect of CAT-8015 on the MDP of RBCs from healthy individuals (*n* = 7). **(B)** Effect of CAT-8015 on the MDP of RBCs from ALL patients (*n* = 9). Control (°); CAT-8015 (▾). Significant differences are indicated (^**^P < 0.01, ^***^P < 0.001, ^****^P < 0.0001). Statistical significance was determined using Holm-Sidak method, with alpha = 0.05, multiple *t*-tests, one unpaired *t*-test per row. At least 50 cells per treatment were analyzed.

**Figure 2:**
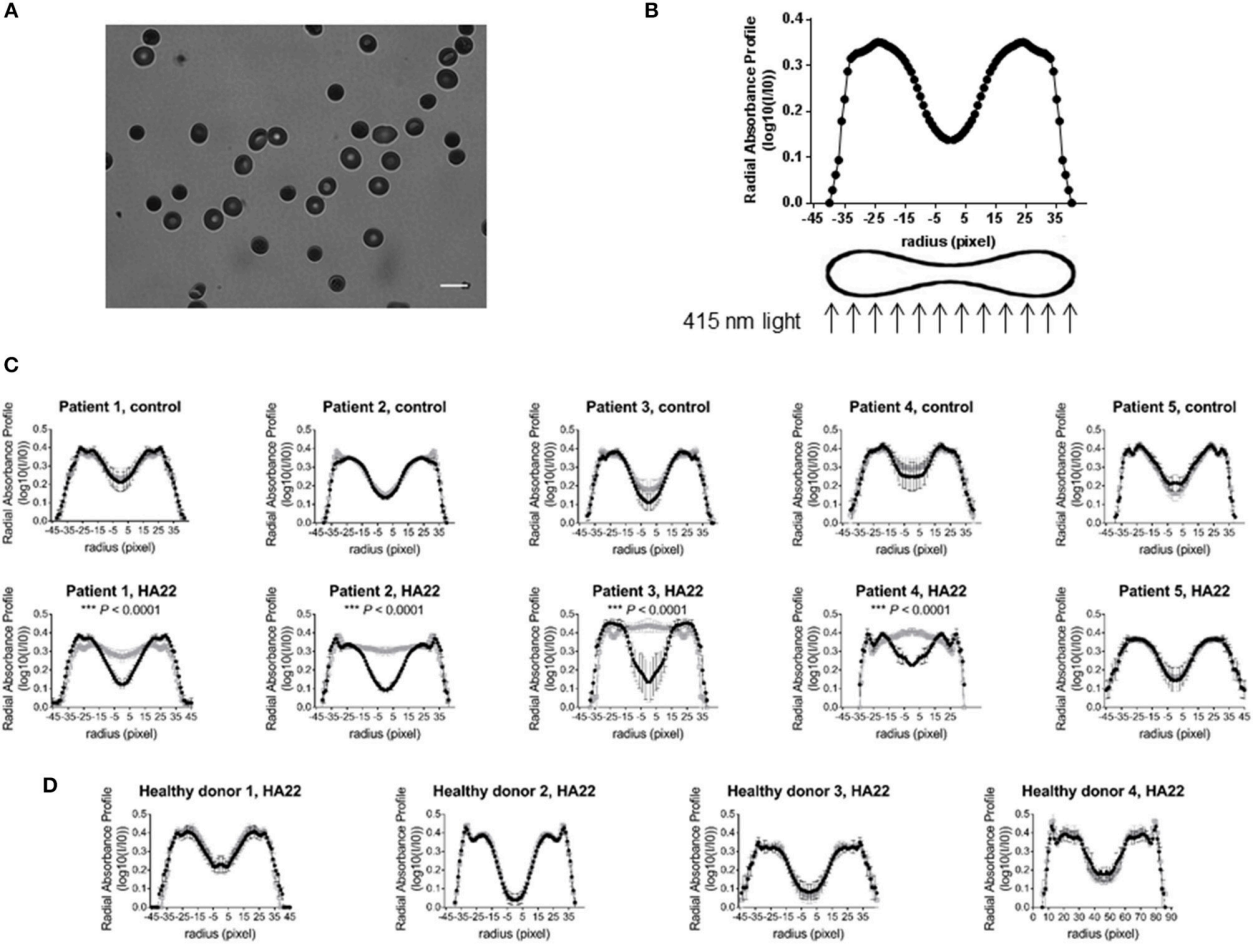
CAT-8015-induced changes in RBC morphology. **(A)** Representative image of RBCs from a pediatric ALL patient exposed to 415 nm light at room temperature in DPBS/BSA buffer. The light regions indicate low absorption of light, while the dark regions indicate high absorption of light. Bar represents 10 μm. **(B)** Representative radial profile of a RBC with a discoid shape. To mimic the shape of RBCs, we plotted the radial absorbance profile of individual cells within each population and mirrored this dataset on the y axis. **(C)** Average radial profiles of RBCs from pediatric ALL patients (*n* = 5) before (in black) and after (in gray) treatment with buffer only (top row), or before (in black) and after (in gray) treatment with buffer containing CAT-8015 (bottom row). **(D)** Average radial profiles of RBCs from healthy donors (*n* = 4) before (in black) and after (in gray) treatment with buffer containing CAT-8015. Statistical significance was determined using multiple t tests. Significant (^***^P < 0.0001) morphological changes in the center of the cell (radius (pixels)−5-5) were evident in ALL patients 1–4 after treatment with CAT-8015. At least 10 cells per treatment were analyzed.

The MDP arises from the orientation and distribution of the dipolar residues of the lipid head groups and the water molecules around them near the surface of the membrane (35), changes in which reveals interaction of molecules with the membrane. To probe CAT-8015 interaction with RBCs, we measured CAT-8015-induced changes in the MDP of the RBC plasma membrane from healthy individuals (*n* = 7) and ALL patients (*n* = 9). The results are shown in Figure 1, where 3 out of 7 healthy individuals (Figure 1A) and 2 out of 9 ALL patients (Figure 1B) showed significant reduction in the MDP of the RBC plasma membrane after CAT-8015 treatment, providing evidence that CAT-8015 disturbs the phospholipid organization of the RBC plasma membrane in susceptible individuals.

### CAT-8015-induced changes in RBC morphology

In separate experiments, and RBCs from different donors, we monitored CAT-8015-induced changes in RBC morphology, determined by measuring the absorption of 415 nm light transmitted through the RBC by hemoglobin. Hemoglobin is uniformly distributed through the cytoplasm of RBCs. Therefore, absorption of 415 nm light by hemoglobin is proportional to the thickness of hemoglobin present in the beam path. In a RBC with a discoid shape absorption of the 415 nm light is lowest in the center and outer edges of the cell, where the cell is thinnest, and highest in the thickest part of the cell. Thus, RBCs with a discoid shape appear as a dark ring with a brighter center and outer edge when exposed to violet light (Figure 2A). The discoid shape of a RBC is reflected in its radial profile, as illustrated in Figure 2B. Figure 2C shows the average radial profiles of individual RBCs from pediatric ALL patients (*n* = 5) before and after exposure to buffer only (top) or buffer containing CAT-8015 (bottom) for 1 h at room temperature. RBCs showed significantly increased absorption of 415 nm light in the center of the cell in 4 out of 5 patients after incubation with CAT-8015, while control cells showed no change in their morphology. We did not observe RBC morphology change in healthy donor samples (*n* = 4) after treatment with buffer containing CAT-8015 (Figure 2D). These data provide evidence that the response to CAT-8015 is ALL specific.

### CAT-8015-induced RBC lysis

**Figure 3:**
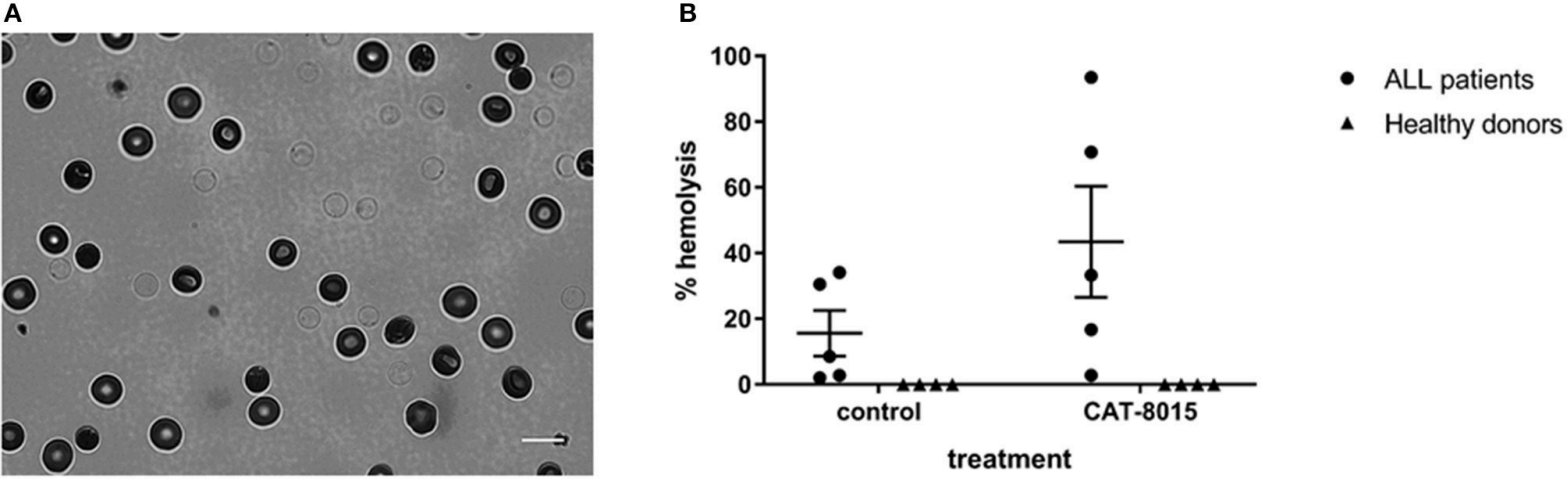
CAT-8015-induced lysis of RBCs from ALL patients**. (A)** A typical 415 nm light image of Di-8-ANEPPS-labeled RBCs from ALL patients after incubation with CAT-8015 for 1 h at room temperature. Lysed cells appear with faint outlines of their membranes, while live cells appear with a halo around their edge. Bar represents 10 μm. **(B)** Haemolysis profiles of Di-8-ANEPPS-labeled RBCs from ALL patients (*n* = 5) and healthy donors (*n* = 4) after incubation for 1 h at room temperature with buffer only (control) or buffer containing CAT-8015. The % lysed cells after each treatment was determined by counting individual RBCs within each population before and after incubation with buffer only (control) or buffer containing CAT-8015. At least 30 cells per treatment were analyzed.

Measuring the absorption of 415 nm light through RBCs also allowed us to monitor CAT-8015-induced lysis of RBCs from the same set of pediatric ALL patients (*n* = 5*)* and healthy donors (*n* = 4*)* that were analyzed for changes in RBC morphology. When exposed to violet light, lysed RBCs appear with faint outlines of their membranes, while live RBCs have a distinctive halo around their edges (Figure 3A). To quantify CAT-8015-induced haemolysis, we counted individual RBCs within each population before and after incubation with buffer only or buffer containing CAT-8015 and calculated the % lysed cells after each treatment. The haemolysis data of RBCs from ALL patients is summarized in Figure 3B. ALL patient samples varied in their sensitivity to CAT-8015, ranging from 3 to 94% cell lysis (mean 43% ± 17 SEM). We also observed RBC lysis in ALL patient samples even after treatment with buffer only, although to a lesser extent compared to after treatment with buffer containing CAT-8015, ranging from 2 to 34% cell lysis (mean 16% ± 7 SEM). We observed no RBC lysis in healthy donor samples after treatment with buffer only or buffer containing CAT-8015 (Figure 3B).

### Correlation between the MDP of RBCS and their susceptibility to CAT-8015-induced lysis

**Figure 4:**
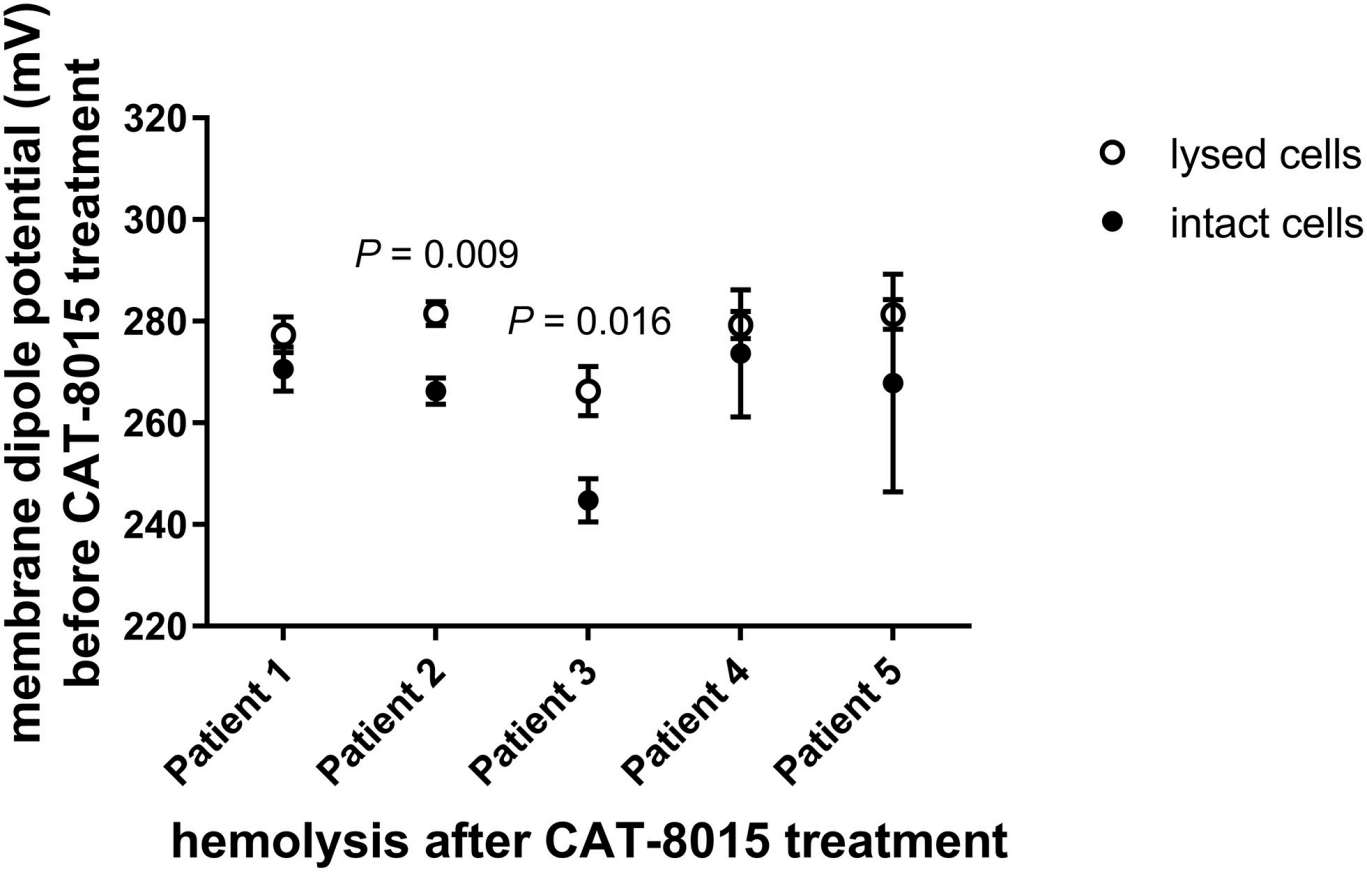
Differences in the MDP of individual cells within a population of RBCs are correlated with their susceptibility to CAT-8015-induced haemolysis. Average (± SEM) MDP values of a population of RBCs from pediatric ALL patients (*n* = 5) before CAT-8015 treatment. Intact cells refer to RBCs that were resistant to CAT-8015-induced lysis while lysed cells refer to RBCs that were sensitive to CAT-8015-induced lysis. Significant differences are indicated. Statistical significance was determined using Holm-Sidak method, with alpha = 0.05, multiple *t*-tests, one unpaired *t*-test per row. At least 20 cells per treatment were analyzed.

We have previously reported that the physical properties of the plasma membrane can influence the susceptibility of RBCs to toxin-induced haemolysis (27), which motivated us to investigate whether differences in the MDP of individual cells within a population of RBCs are correlated with their susceptibility to CAT-8015-induced lysis. This involved measuring the MDP for a number of individual cells within a population of RBCs before CAT-8015 treatment in the same set of pediatric ALL patients (*n* = 5) as shown in Figures 2, 3. RBCs were then exposed to 415 nm light after 1 h treatment with CAT-8015 at room temperature to monitor haemolysis. Figure 4 shows that in all patients tested RBCs with prolonged resistance to CAT-8015 treatment (intact cells) have lower pre-treatment MDP values relative to cells with increased sensitivity to CAT-8015 treatment (lysed cells), with significant differences in 2 patients, indicating that higher MDP values are correlated with increased sensitivity to CAT-8015-induced lysis.

### Interaction of CAT-8015 with RBCs is *via* its toxin component, PE38

**Figure 5:**
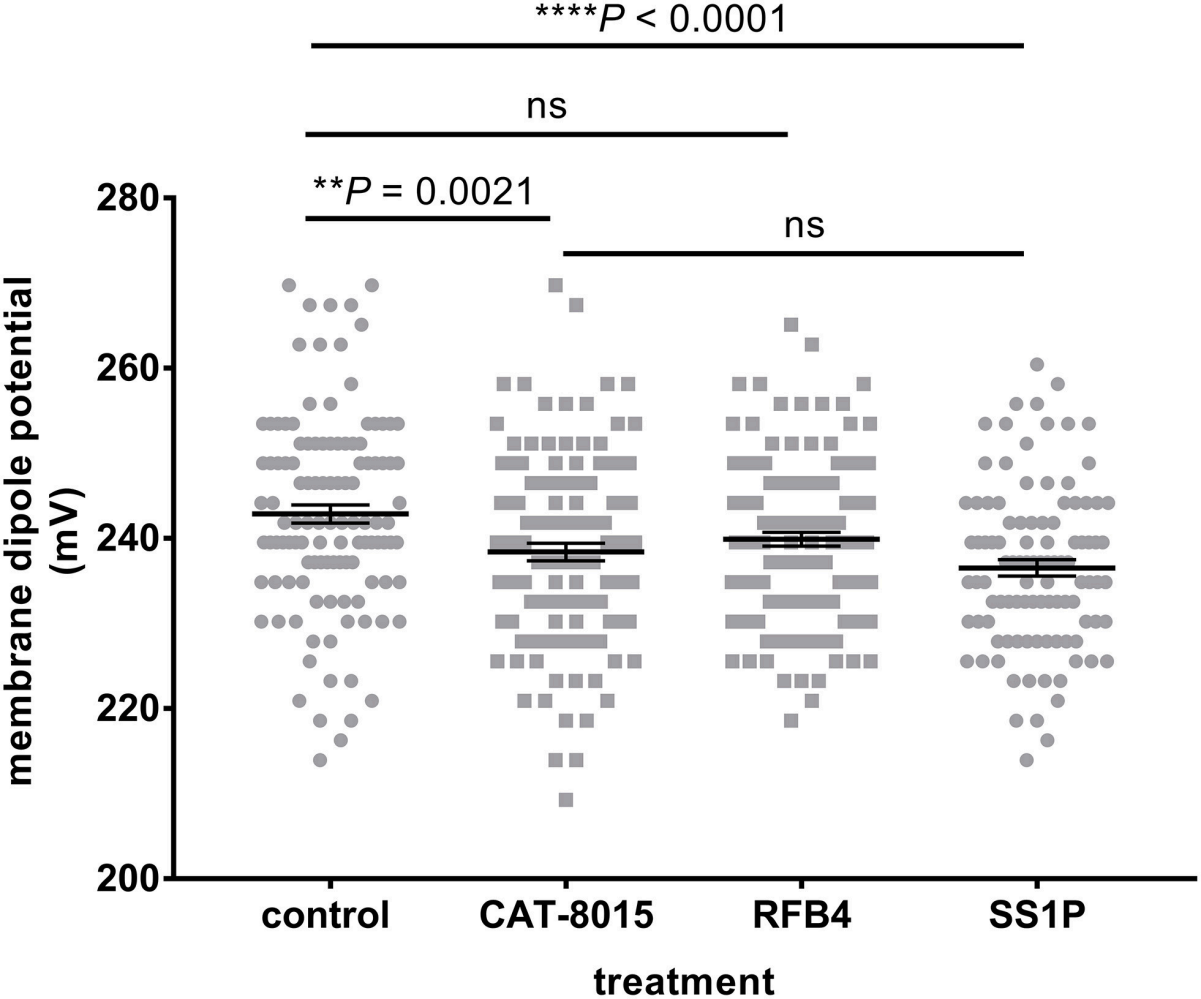
Interaction of CAT-8015 with RBCs is via its toxin component, PE38. Representative dot plot of changes in the MDP of Di-8-ANEPPS-labelled RBCs from healthy individuals (*n* = 3) after treatment with buffer only (control) or buffer containing CAT-8015, RFB4 (an anti-CD22 monoclonal antibody to mimic the antibody component of CAT-8015) or SS1P (an anti-mesothelin *Pseudomonas* exotoxin A-based immunotoxin to mimic the toxin component of CAT-8015). Each dot represents an individual cell within a population of RBCs. At least 100 cells per treatment were analysed. Statistical significance was determined using Ordinary One-Way ANOVA, Dunnett multiple comparison test. Significant differences are indicated (^**^p = 0.0021, ^****^p < 0.0001), ns, not significant. One representative experiment out of three is shown.

To investigate which component of CAT-8015 is responsible for interaction with RBCs, we incubated Di-8-ANEPPS-labeled RBCs from healthy individuals (*n* = 3) previously shown to be susceptible to CAT-8015 treatment (Figure 1A) with buffer only or buffer containing CAT-8015, RFB4 (an anti-CD22 monoclonal antibody to mimic the antibody component of CAT-8015) or SS1P (an anti-mesothelin *Pseudomonas* immunotoxin to mimic the toxin component of CAT-8015). Figure 5 shows a representative dot plot of a population of RBCs after the above treatments. RBCs treated with SS1P showed significant reduction in their MDP values relative to control cells, similar to RBCs treated with CAT-8015, while RBCs treated with RFB4 showed MDP values similar to control cells, indicating that CAT-8015 interacts with RBCs *via* its toxin component. RBCs treated with SS1P showed MDP values similar to cells treated with CAT-8015.

### Human mesentery tissue, an *ex vivo* EC model to investigate interaction of CAT-8015 with vascular ECs

**Figure 6:**
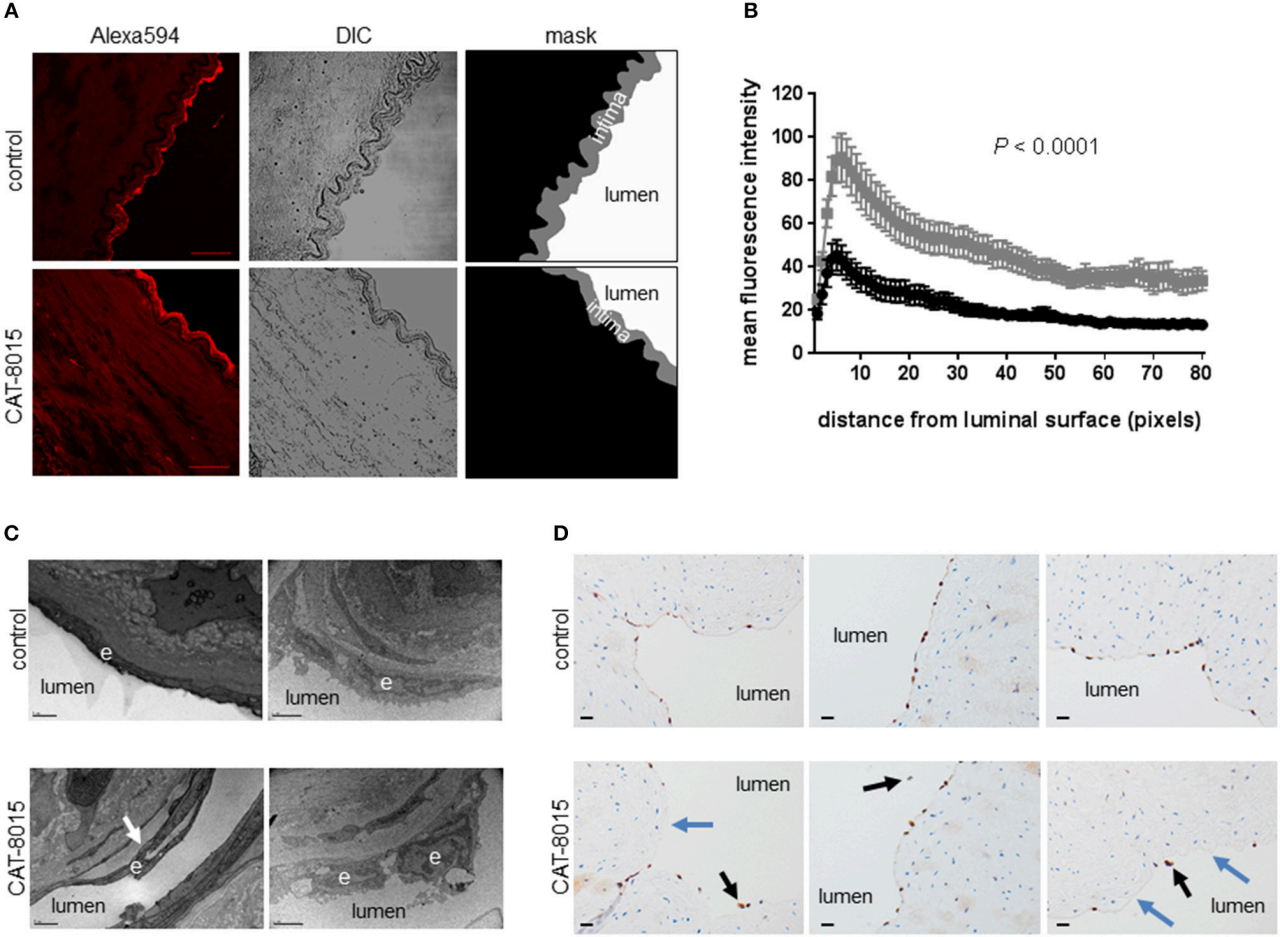
The effect of CAT-8015 on ECs of excised human mesenteric arteries. **(A)** Representative confocal microscope images of transverse cryosections (10 μm) of excised human mesenteric arteries (*n* = 3) incubated with buffer only (top) or buffer containing CAT-8015 (bottom) for 1 hour at 37°C in the presence of the vascular permeability marker Alexa594-BSA. Bars represent 40,μm. To quantify the effect of CAT-8015 on vascular permeability, Alexa594 images (left) were converted to mask images (right) and the mean intensity of red fluorescence across the intima (in grey) was plotted against the distance from the luminal surface (expressed in pixels, 1 pixel = 0.5 μm). **(B)** Average (± SEM) profiles of red fluorescence from excised human mesentery arteries (*n* = 3) treated with buffer only (in black) or buffer containing CAT-8015 (in gray). Image analysis was performed for 10–15 different images per sample. Statistical significance was determined using unpaired, two-tailed *t*-test with Welch's correction. **(C)** Representative transmission electron microscopy images of cross-sections of excised human mesenteric arteries (*n* = 2) incubated for 1 h at 37°C with buffer only (control) or buffer containing CAT-8015. Arrows indicate detachment of ECs (e) from the basal membrane. Bars represent 1 μm (left), and 2 μm (right). **(D)** Representative ERG immunohistochemistry images of cross-sections of excised human mesenteric arteries (*n* = 3) incubated for 1 h at 37°C with buffer only (control) or buffer containing CAT-8015. Black arrows indicate detached ECs from the basal membrane while blue arrows indicate discontinuous EC surfaces in CAT-8015-treated vessels. The ECs lining vessel lumens in control vessels were continuous. Bars represent 20 μm.

To investigate interaction of CAT-8015 with vascular ECs, we established an *ex vivo* EC model using excised human mesenteric arteries. To investigate the effect of CAT-8015 on vascular permeability, we incubated arteries (*n* = 3) with buffer only or buffer containing CAT-8015 in the presence of the vascular permeability marker albumin labeled with the red fluorescent dye Alexa Fluor™ 594 for 1 hour at 37°C, and cryosections of arteries were imaged by confocal microscopy (Figure 6A). To quantify the effect of CAT-8015 on vascular permeability, images were converted to mask images (Figure 6A, bottom) and the mean intensity of red fluorescence across the intima (in gray), the innermost layer of the artery wall that contains a monolayer of ECs, was plotted against the distance from the luminal surface. As illustrated in Figure 6B, there is significantly increased red fluorescence below the endothelial surface of vessels treated with CAT-8015 compared to control vessels, indicating that CAT-8015 increased vascular permeability to protein.

### Ultrastructural and histological evaluation of the effect of CAT-8015 on vascular ECs

To further evaluate the effect of CAT-8015 on vascular ECs, we incubated excised human mesenteric arteries with buffer only or buffer containing CAT-8015, and after incubation for 1 h at 37°C tissues were processed for ultrastructural and histological evaluation. Figure 6C shows representative TEM images of artery cross-sections from two experiments. We observed differences between untreated vessels and vessels treated with CAT-8015, which included CAT-8015-induced detachment of ECs from the basement membrane (Figure 6C, bottom), consistent with damage to the vasculature that could allow for protein and fluid leak. Control vessels showed normal cell morphology (Figure 6C, top). Light microscopy of H&E-and ERG-stained histological sections of human mesenteric arteries also revealed detachment of ECs from the basement membrane after CAT-8015 treatment (Figure 6D, bottom), with endothelial discontinuities frequently seen. In contrast,ECs lining vessel lumens were continuous and without gaps in untreated vessels (Figure 6D, top).

## Discussion

The pathogenesis of CAT-8015-induced HUS/VLS is not well understood due to the lack of an adequate animal model system that closely corresponds to clinical manifestations. Therefore, the present study developed human models and used novel methodologies to investigate interactions of CAT-8015 with red blood cells (RBCs) from pediatric ALL patients and ECs of excised human mesenteric arteries. In all our experiments we used RBCs and tissues after plasma had been removed and the cells and tissues were washed extensively to remove complement.

Our study, for the first time, provides evidence that CAT-8015 directly interacts with RBCs, mediated by its toxin component PE38, a novel mechanism for the pathogenesis of atypical HUS. Interaction of CAT-8015 with RBCs was also independent of platelet-induced haemolysis or RBC fragmentation, the latter thought to be the consequence of high levels of shear stress in obstructed vessels due to thrombosis (11–13). Although the focus of our study was a *Pseudomonas* IT, our study has wider applicability to other toxins and ITs that cause atypical HUS, such as Shiga toxin (11), Combotox®, a ricin-based IT targeting CD19 and CD22 (36), and DAB_486_IL-2, a diphtheria toxin-based IT targeting interleukin-2 (37, 38).

Measurements of the MDP and RBC morphology revealed interpatient variability in response to CAT-8015 treatment. Considerable interpatient variability in CAT-8015-induced cytotoxicity was also observed in a study of 35 primary ALL patient samples (39, 40). Measurements of the absorption of 415 nm light by hemoglobin also revealed that in ALL patients RBCs lyse even after treatment with buffer only. This observation is in line with a small number of case reports in which diagnoses of ALL and HUS were made prior to initiation of any anti-leukemic treatment, suggesting that in ALL the disease itself may trigger RBC lysis (13).

We have previously reported that the physical properties of the plasma membrane can influence cell susceptibility to protein toxins, where higher MDP values correlated with reduced time to toxin-induced lysis (27). In the present study we found a similar correlation between the electrical properties of the RBC membrane and RBC susceptibility to CAT-8015-induced lysis, where RBCs with higher MDP values showed increased sensitivity to CAT-8015-induced lysis. This may have implications in clinical medicine. Measurements of the physical properties of the RBC membrane are straightforward and could identify patients who will most benefit from CAT-8015 immunotoxin therapy.

Measurements of the MDP indicate that interaction of CAT-8015 with RBCs is *via* its toxin component, PE38. It is possible that CAT-8015 contains a site which could be recognized with low affinity by RBCs, thus accounting for haemolysis. Weldon et al. (41). reported on a variant of CAT-8015 (HA22-LR) that has a deletion of a large portion of the membrane translocation domain II of *Pseudomonas* exotoxin A that is fully cytotoxic, with greatly diminished non-specific toxicities in mice while preserving on-target cytotoxicity, which may be due to loss of residues that interact with RBCs and cause membrane damage, and possibly HUS in patients. Our preliminary studies also indicate that HA22-LR is less toxic to RBCs compared to CAT-8015 (data not shown). Further studies will be required to determine the minimal sequence responsible for PE-induced RBC lysis that may lead to HUS.

While the interaction of CAT-8015 with the RBC membrane might be relatively weak, it has a number of important practical implications: (1) CAT-8015 will come into contact with a very large number of RBCs so even weak binding could influence its kinetics and availability in the circulation; (2) in an individual, all plasma membranes have similar lipid composition so these interactions may well be involved in the vascular responses; (3) our previous work on other toxins indicates that such interactions are influenced by factors, such as oxidative stress (27), which may be involved in interpatient variability in response to CAT-8015 treatment. Interpatient variability in response to CAT-8015 treatment may also be linked to additional factors, such as treatment timing, initial burden, immune response, variation in the kinetics of internalization and turnover between patient samples, and efficiency of intracellular processing (42, 43). The weak and transient interaction between CAT-8015 and the RBC membrane may trigger changes in RBC morphology and induce haemolysis, which may account for HUS in patients. However, the role of CAT-8015 interaction with RBCs in HUS pathogenesis remains to be more rigorously explored. Future studies could include classic receptor-ligand binding analyses to measure binding affinities and on/off rates.

Our study is also the first to report that CAT-8015 is directly cytototoxic to ECs of excised human mesenteric arteries, evidenced by increased vascular permeability and detachment of ECs from the basement membrane, which may account for VLS in patients. Although PE in its native form also causes EC damage (44), CAT-8015 is not expected to show cytotoxic activity toward ECs but only to cells expressing the CD22 antigen. It is possible that PE contains, in addition to the specific cell-binding domain I which is removed in CAT-8015, an additional site which could be recognized with low affinity by ECs, thus accounting for VLS in patients. Several studies suggest that induction of VLS requires an enzymatically active toxin molecule (45, 46). One study suggests that toxins that contain a three-amino acid consensus motif in their enzymatically active domain may be responsible for binding to ECs and initiating VLS in humans (47). Subsequent studies showed that modification or deletion of this motif reduced toxin-induced VLS (48, 49). Further studies will be required to determine the minimal sequence responsible for PE-induced EC damage that may lead to VLS.

CAT-8015 may directly contribute to HUS/VLS by inhibiting protein synthesis in vascular ECs, similar to Shiga toxin mediated VLS/HUS (50). Additional studies could explore the use of isolated glomerular tufts to investigate the role of CAT-8015-mediated damage of glomerular ECs in the development of HUS/VLS, such as by exploring the relationship between protein synthesis inhibition and EC cytotoxicity using enzymatically inactive PE-antibody constructs. Induction of VLS also requires the membrane translocation domain II of PE, since the protease-resistant variant of CAT-8015 (HA22-LR) with a large deletion in domain II of PE can diminish non-specific toxicity in mice, which may be due to loss of residues that interact with ECs and cause liver damage in mice, and possibly VLS in patients (4, 41). Alternatively, CAT-8015 may indirectly contribute to VLS/HUS by inducing release of cytokines from damaged vascular endothelium (16). It is possible that nonspecific binding of CAT-8015 fragments to ECs and/or blood cells cause VLS/HUS upon release from the target cells.

In conclusion, the human models we developed in this study constitutes the first, and very important, step in understanding the origins of HUS/VLS in immunotherapy and will allow further investigations of HUS/VLS pathogenesis. The present study also provides new biophysical tools: (1) to evaluate specific drugs for their ability to limit the side effects of *Pseudomonas* IT therapy in pediatric ALL patients; (2) to develop novel drugs that avoid HUS/VLS; and (3) to study complement-independent HUS caused by other toxins and ITs. The *ex vivo* human mesentery model we developed in this study could also be exploited in future studies to correlate the observed changes in RBC association and vascular leak to the development of HUS/VLS.

## Author contributions

MB-B performed experiments, developed methodology, analyzed results, made the figures, and wrote the paper. JM wrote the image processing scripts and performed some of the image analysis. CW and PP developed experimental methods. CW, PP, RT, FM, and MB-B conceived the idea for the project, designed the research, coordinated the study and revised the manuscript. BK and CD recruited participants and acquired samples. NS made contributions to the design of the research and acquired samples. SS made contributions to the design of the research, acquired samples, and made contributions to Figure 6. IP provided purified immunotoxins. All authors reviewed the results and approved the final version of the manuscript.

### Conflict of interest statement

The authors declare that the research was conducted in the absence of any commercial or financial relationships that could be construed as a potential conflict of interest.
